# A community-based transcriptomics classification and nomenclature of neocortical cell types

**DOI:** 10.1038/s41593-020-0685-8

**Published:** 2020-08-24

**Authors:** Rafael Yuste, Michael Hawrylycz, Nadia Aalling, Argel Aguilar-Valles, Detlev Arendt, Ruben Armañanzas, Giorgio A. Ascoli, Concha Bielza, Vahid Bokharaie, Tobias Borgtoft Bergmann, Irina Bystron, Marco Capogna, YoonJeung Chang, Ann Clemens, Christiaan P. J. de Kock, Javier DeFelipe, Sandra Esmeralda Dos Santos, Keagan Dunville, Dirk Feldmeyer, Richárd Fiáth, Gordon James Fishell, Angelica Foggetti, Xuefan Gao, Parviz Ghaderi, Natalia A. Goriounova, Onur Güntürkün, Kenta Hagihara, Vanessa Jane Hall, Moritz Helmstaedter, Suzana Herculano-Houzel, Markus M. Hilscher, Hajime Hirase, Jens Hjerling-Leffler, Rebecca Hodge, Josh Huang, Rafiq Huda, Konstantin Khodosevich, Ole Kiehn, Henner Koch, Eric S. Kuebler, Malte Kühnemund, Pedro Larrañaga, Boudewijn Lelieveldt, Emma Louise Louth, Jan H. Lui, Huibert D. Mansvelder, Oscar Marin, Julio Martinez-Trujillo, Homeira Moradi Chameh, Alok Nath Mohapatra, Hermany Munguba, Maiken Nedergaard, Pavel Němec, Netanel Ofer, Ulrich Gottfried Pfisterer, Samuel Pontes, William Redmond, Jean Rossier, Joshua R. Sanes, Richard H. Scheuermann, Esther Serrano-Saiz, Jochen F. Staiger, Peter Somogyi, Gábor Tamás, Andreas Savas Tolias, Maria Antonietta Tosches, Miguel Turrero García, Christian Wozny, Thomas V. Wuttke, Yong Liu, Juan Yuan, Hongkui Zeng, Ed Lein

**Affiliations:** 1 Columbia University, New York City, NY USA; 2 Allen Institute for Brain Science, Seattle, WA USA; 3 University of Copenhagen, Copenhagen, Denmark; 4 Department of Neuroscience, Carleton University, Ottawa, Ontario Canada; 5 European Molecular Biology Laboratory, Heidelberg, Germany; 6 George Mason University, Fairfax, VA USA; 7 BrainScope Company Inc., Bethesda, MD USA; 8 Universidad Politécnica de Madrid, Madrid, Spain; 9 Max Planck Institute for Biological Cybernetics, Tübingen, Germany; 10 University of Oxford, Oxford, UK; 11 Department of Biomedicine, Aarhus University, Aarhus, Denmark; 12 Department of Genetics, Harvard Medical School, Boston, MA USA; 13 The University of Edinburgh, Edinburgh, UK; 14 Vrije Universiteit Amsterdam, Amsterdam, Netherlands; 15 Cajal Institute, Madrid, Spain; 16 Vanderbilt University, Nashville, TN USA; 17 Scuola Normale Superior, Pisa, Italy; 18 Research Centre Jülich, Jülich, Germany; 19 Research Centre for Natural Sciences, Budapest, Hungary; 20 Harvard Medical School, Cambridge, MA USA; 21 Christian-Albrechts-University Kiel, Kiel, Germany; 22 European Molecular Biology Laboratory, Hamburg, Germany; 23 École Polytechnique Fédérale de Lausanne, Lausanne, Switzerland; 24 Ruhr University Bochum, Bochum, Germany; 25 Friedrich Miescher Institute for Biological Research, Basel, Switzerland; 26 Max Planck Institute for Brain Research, Frankfurt, Germany; 27 Karolinska Institutet, Stockholm, Sweden; 28 Science for Life Laboratory, Department of Biochemistry and Biophysics, Stockholm University, Solna, Sweden; 29 Cold Spring Harbor Laboratory, Laurel Hollow, NY USA; 30 WM Keck Center for Collaborative Neuroscience, Department of Cell Biology and Neuroscience, Rutgers University - New Brunswick, Piscataway, NJ USA; 31 Department of Neuroscience, University of Copenhagen, Copenhagen, Denmark; 32 RWTH Aachen University, Aachen, Germany; 33 Robarts Research Institute, Western University, London, Ontario Canada; 34 CARTANA, Stockholm, Sweden; 35 Leiden University Medical Center, Leiden, the Netherlands; 36 Stanford University, Stanford, CA USA; 37 King’s College London, London, UK; 38 Schulich School of Medicine and Dentistry, Departments of Physiology, Pharmacology and Psychiatry, University of Western Ontario, London, Ontario Canada; 39 Krembil Research Institute, Toronto, Ontario Canada; 40 University of Haifa, Haifa, Israel; 41 Univeristy of Rochester, Rochester, NY USA; 42 Charles University, Prague, Czech Republic; 43 Bar Ilan University, Ramat Gan, Israel; 44 Macquarie University, Sydney, New South Wales Australia; 45 Sorbonne University, Paris, France; 46 Harvard University, Cambridge, MA USA; 47 J. Craig Venter Institute, La Jolla, CA USA; 48 Department of Pathology, University of California, San Diego, CA USA; 49 Centro de Biologia Molecular Severo Ochoa (CSIC), Madrid, Spain; 50 Institute for Neuroanatomy, University of Göttingen, Göttingen, Germany; 51 University of Szeged, Szeged, Hungary; 52 Baylor College of Medicine, Houston, TX USA; 53 Department of Neurobiology, Harvard Medical School, Boston, MA USA; 54 University of Strathclyde, Glasgow, UK; 55 MSH Medical School, Hamburg, Germany; 56 Departments of Neurosurgery and of Neurology and Epileptology, Hertie-Institute for Clinical Brain Research, University of Tübingen, Tübingen, Germany

**Keywords:** Genetics of the nervous system, Neural circuits

## Abstract

To understand the function of cortical circuits, it is necessary to catalog their cellular diversity. Past attempts to do so using anatomical, physiological or molecular features of cortical cells have not resulted in a unified taxonomy of neuronal or glial cell types, partly due to limited data. Single-cell transcriptomics is enabling, for the first time, systematic high-throughput measurements of cortical cells and generation of datasets that hold the promise of being complete, accurate and permanent. Statistical analyses of these data reveal clusters that often correspond to cell types previously defined by morphological or physiological criteria and that appear conserved across cortical areas and species. To capitalize on these new methods, we propose the adoption of a transcriptome-based taxonomy of cell types for mammalian neocortex. This classification should be hierarchical and use a standardized nomenclature. It should be based on a probabilistic definition of a cell type and incorporate data from different approaches, developmental stages and species. A community-based classification and data aggregation model, such as a knowledge graph, could provide a common foundation for the study of cortical circuits. This community-based classification, nomenclature and data aggregation could serve as an example for cell type atlases in other parts of the body.

## Classifications of cortical cell types: from Cajal to the Petilla Convention

The conceptual foundation of modern biology is the cell theory of Virchow, which described the cell as the basic unit of structure, reproduction and pathology of biological organisms^1^. This idea, which arose from the use of microscopes by Leeuwenhoek, Hooke, Schleiden and Schwann, among others, generated the need to build catalogs of the cellular components of tissues as the first step toward studying their structure and function. As with species, these cell catalogs, or atlases, can be ideally systematized into ‘cell taxonomies’, classifying groups of cells based on shared characteristics and grouping them into taxa with ranks and a hierarchy. Taxonomies are important: they provide a conceptual foundation for a field and also enable the systematic accumulation of knowledge. Essential to this effort is the clear definition of cell type, normally understood as cells with shared phenotypic characteristics.

Virchow’s cell theory was introduced to neuroscience by Cajal, whose ‘neuron doctrine’ postulated that the structural unit of the nervous system was the individual neuron^2^. Since then, generations of investigators have described hundreds of cell types in nervous systems of different species. This effort has been particularly arduous in the cerebral cortex (or neocortex), the largest part of the brain in mammals and the primary site of higher cognitive functions. The mammalian neocortex has a thin layered structure, composed of mixtures of excitatory and inhibitory neurons arranged in circuits of a forbidding complexity, called “impenetrable jungles” by Cajal^3^. This basic structure is very similar in different cortical areas and in different species, which has given rise to the possibility that there is a ‘canonical’ cortical microcircuit^4–7^, replicated during evolution, which underlies all cortical function.

**Fig. 1:**
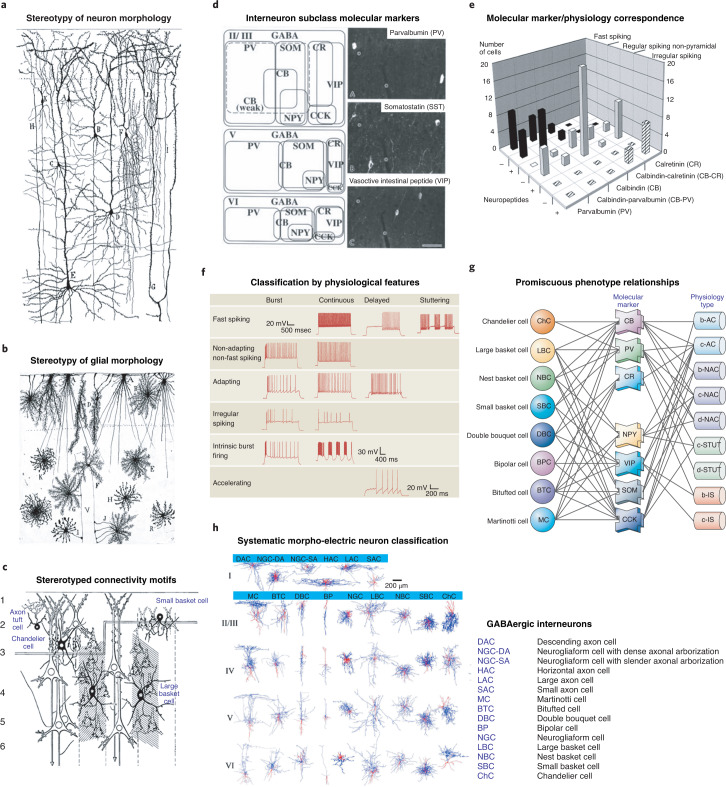
Non-transcriptomics cortical cell-type classifications. **a**,**b**, Morphological characterization and classification of neurons (**a**) and glial cells (**b**) by Ramón y Cajal (1904)^4^. **c**, Diagram showing the connections of different types of interneurons with pyramidal cells. Adapted from Szentágothai (1975)^9^. **d**, Definition of GABAergic interneuron classes based on non-overlapping and combinatorial marker gene expression. **e**, Correlation of firing properties with class markers. **f**, Cortical cell type classification based on intrinsic firing properties (Petilla convention). **g**, Complex relationships between cellular morphology, marker-gene expression and intrinsic firing properties based on multimodal analysis. **h**, Comprehensive morphological and physiological classifications of cortical cell types. Images in **a**,**b** reprinted with permission from ref. ^4^, Cajal Institute; in **c**, adapted with permission from ref. ^9^, Elsevier; in **d**, adapted with permission from ref. ^25^, Oxford Univ. Press; in **e**, adapted with permission from ref. ^14^, Society for Neuroscience; in **f** and **g**, adapted with permission from refs. ^17,21^, respectively, Springer Nature; in **h**, adapted with permission from ref. ^23^, Cell Press.

After more than a hundred years of sustained progress, it is clear that neocortical neurons and glial cells, like cells in any tissue, belong to many distinct types. Different cell types likely play discrete roles in cortical function and computation, making it important to characterize and describe them accurately and in their absolute and relative numbers. Towering historical figures like Cajal, Lorente de Nó and Szentágothai, among others, proposed classifications of cortical cells based on their morphologies as visualized with histological stains^4,8,9^ (Fig. 1a–c). These anatomical classifications described several dozen types of pyramidal neurons, short-axon cells and glial cells, and they were subsequently complemented by morphological accounts of additional cortical cell types by many researchers^10–12^, but without arriving at a clear consensus as to the number or even the definition of a cortical cell type.

Over the last few decades, the introduction of new morphological, ultrastructural, immunohistochemical and electrophysiological methods, new molecular markers, and a growing appreciation of the developmental origins of distinct neuronal subtypes (Fig. 1d–h), have provided increasingly finer phenotypic measurements of cortical cells and enabled new efforts to classify them more quantitatively, using supervised or unsupervised methods such as cluster analysis^13–16^. A community effort to classify neocortical inhibitory cells was attempted at the 2005 Petilla Convention, held in Cajal’s hometown in Spain, and led to a common standardized terminology describing the anatomical, physiological and molecular features of neocortical interneurons^17^. While useful, this fell short of providing a classification and working framework that investigators could incorporate into their research. One reason why this early effort failed was because the datasets for phenotypically characterizing cortical neurons were small. Indeed, many of the early studies are based on characterizing dozens or at most hundreds of neurons, small samples from the nearly 20 billion in human neocortex^18^.

An outcome of the Petilla Convention was the realization that there was not yet a single method that captured the inherently multimodal nature of cell phenotypes and could serve as a standard for classification. While most researchers accepted the existence of cell types that could be measured and defined independently by different methods, there was no agreement as to which would form an optimal basis for classification. In principle, many criteria can be used, including (i) anatomical or connectivity-based features^19,20^, (ii) parametrization of intrinsic electrophysiological properties^21^, (iii) combination of structural and physiological criteria^22,23^, (iv) molecular markers^14,24,25^, (v) developmental origins^26,27^, (vi) epigenetic attractor states^28^ or (vii) evolutionary approaches identifying homology across species^29,30^. Ideally, these classifications should converge and agree, or at least substantially overlap. Indeed, there is substantial concordance among categories based on anatomical, molecular and physiological criteria^13,22,31–34^, but it has not been easy to combine these approaches into a unified taxonomy. There are substantial differences between researchers in assigning neurons to particular types in the literature^19^, and even experts often disagree on what constitutes ground truth. For example, while most publications agree on what a chandelier cell is, the concept of basket cells, a major subtype of inhibitory neuron, is much less clear^19^.

This uncertainty is explained and exacerbated by technical challenges: conventional approaches have been laborious, low-throughput, frequently non-quantitative and generally plagued by an inability to sample cells in standardized and systematic ways. Thus, setting aside debates about the importance of various criteria and the nature or even existence of discrete cell types, it is not surprising that the cell-type problem has remained challenging.

## Transcriptomics: a new framework for classifying cortical cell types

**Fig. 2:**
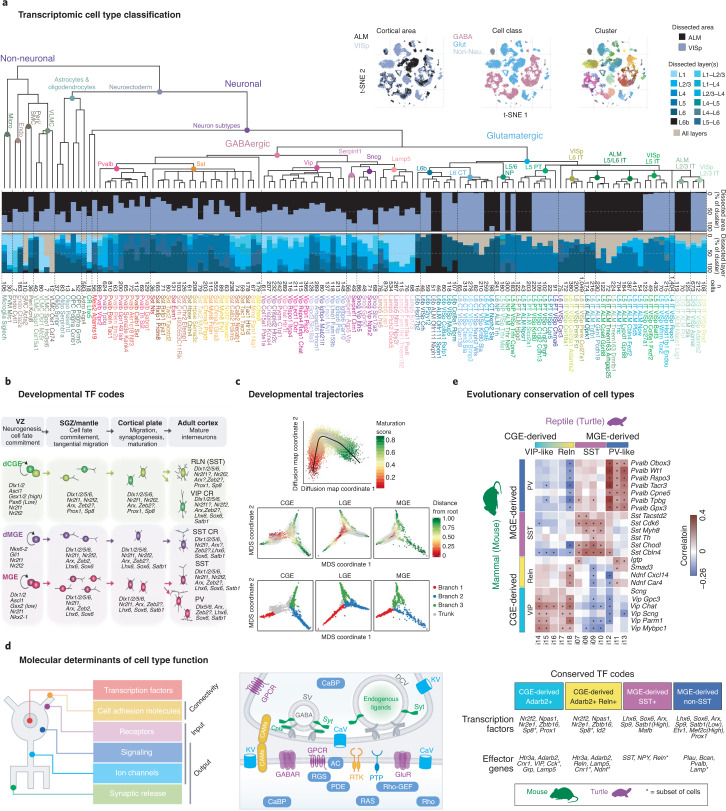
Transcriptomics classifications of cortical cell types. **a**, Single-cell transcriptome analysis reveals a molecular diversity of mouse cell types, with relatively invariant interneuron and non-neuronal types across cortical areas but significant variation in excitatory neurons. **b**, Major interneuron classes are specified by distinct transcription factor codes. **c**, Single-cell transcriptomics of mouse GABAergic interneuron development demonstrates gradual changes in gene expression underlying developmental maturation and fate bifurcations as cells become postmitotic. **d**, Gene families shaping cardinal GABAergic neuron type include neuronal connectivity, ligand receptors, electrical signaling, intracellular signal transduction, synaptic transmission and gene transcription. These gene families assemble membrane-proximal molecular machines that customize input–output connectivity and properties in different GABAergic types. **e**, Single-cell transcriptomics allows cross-species comparisons and shows conservation of major cell classes from reptiles to mammals, with conserved transcription factors but some species-specific effectors (turtle data). TF, transcription factor. Images in **a** and **c** adapted with permission from refs. ^40,63^, respectively, Springer Nature; in **b**, adapted with permission from ref. ^27^, Elsevier; in **d**, adapted with permission from ref. ^37^, Cell Press; in **e**, adapted with permission from refs. ^30,68^, Elsevier and AAAS, respectively.

Recent advances in high-throughput single-cell transcriptomics (scRNAseq) have changed the paradigm of cellular classification, offering a new quantitative genetic framework^35–40^. These approaches measure the expression profiles of thousands of genes from individual cells in large numbers, at relatively high speed and low cost. Related methods in epigenomics can identify sites of methylation and putative gene transcriptional regulation, essential to cell function and state. These new methods are an outcome from the methodological, conceptual and economic revolution created by the Human Genome Project^41^ and have flourished with support from the BRAIN Initiative^42,43^. With genomes in hand, it is now feasible to generate entire transcriptomes (which include the sequence and structure of transcripts) from tissues and to scale these methods for amplifying RNA in single cells. Initially limited to only a few hundred cells per experiment, effective new methods have emerged for profiling thousands of cells or nuclei at a time^44–48^. With simultaneous computational advances for analyzing large sequence-based data^49,50^, it is now possible to systematically classify and characterize the diversity of neural cells in any tissue, including the neocortex (Fig. 2).

Conceptually, as much as the genome is the internal genetic description for each species, the transcriptome, as the complete set of genes being expressed, provides an internal code that can describe each cell within an organism in a spatiotemporal context. Practically, the scale of scRNAseq promises near-saturating analysis of complex cellular brain regions like the neocortex, providing, for the first time, a comprehensive and quantitative description of cellular diversity and the prospect of simplifying tissue cell composition to a finite number of cell types and states defined by statistical clustering. Importantly, however, these transcriptionally defined clusters represent a probabilistic description of cell types in a high-dimensional landscape of gene expression across all cells in a tissue, rather than a definition based on a small set of necessary and sufficient cellular markers or other features (see below).

The scale, precision and information content of these current methods now far outpace other classical methods of cellular phenotyping in neuroscience and have the potential to approach the complete, accurate and permanent (CAP) criteria cited by Brenner as the gold standard in biological science^51^. Indeed, major efforts now aim to generate a complete description of cell types based on molecular criteria across the neocortex (Allen Institute for Brain Science^36,40^), the whole brain (the National Institutes of Health (NIH) BRAIN Initiative Cell Census Network^52^) and even the whole body (the Human Cell Atlas^53^). Also, as the Human Genome Project offered a means for comparative analysis of orthologous genes across species, these efforts could define all or most cell types and states in humans and model organisms, with the possibility of extending them to a variety of species to understand the evolution of cell-type diversity. These large investments have the potential for a transformative effect on neuroscience, which will be accelerated by a formalization of a molecular classification and its adoption by the community. They also hold promise for the development of methods for querying circuit function by providing tools for the targeting and manipulation of particular subtypes.

Transcriptomic classification offers the following advantages as a framework for bounding the problem of cellular diversity^53–56^:

1. High-throughput transcriptomics is very effective at allowing a systematic, comprehensive analysis of cellular diversity in complex tissues. Its quantitative and high-throughput nature enables the adoption of rigorous definitions and criteria using datasets from tens of thousands to millions of cells.
2. The genes expressed by a cell during its development and maturity ultimately underlie its structure and function, and so the transcriptome offers predictive power based on interpreting gene function. Other cellular phenotypes, including morphology, are in part encoded by genes, rather than completely independent defining criteria^57^.
3. A molecular definition of cell types allows the identification of cell-type markers and the creation of genetic tools to target, label and manipulate specific cell types^58,59^, thereby providing the means to standardize datasets obtained by different researchers.
4. Transcriptomic data can also provide information about human diseases, by allowing a potential linkage between genes associated with disease and their cellular locus of action. By combining with genome-wide association studies (GWAS) that identify genes causally involved in the pathophysiology of a disease, cell-type transcriptomics-based data might lead to identification of mechanistically unresolved diseases as detected changes in expression levels of genes from key cell types^60^.
5. Expression profiles allow quantitative comparison of cell types across evolutionary or developmental times, enabling the alignment of cell types across species (based on conserved expression of homologous genes)^61^ and developmental stages (based on gradual developmental trajectories)^62–64^.
6. Transcriptomics also enables comparing cell types across organs, as different organs use similar genes. Thus, it could be used to classify all the cells in the body with a single method and framework^53^.

Indeed, initial transcriptomic studies of cortical tissue are already providing many biological insights. For example, scRNAseq analysis of mouse and human cortex identified a complex but finite set of ~100 molecularly defined cell types per cortical region that generally agree with prior literature on cytoarchitectural organization, developmental origins, functional properties and long-range projections^65^. Moreover, the hierarchical (agglomerative) taxonomy of transcriptomic cell types^66^, based on relative similarity between clusters, reflects these organizational principles. Viewed as a tree or dendrogram, the initial branches reflect major classes (neuronal vs non-neuronal; excitatory vs inhibitory), with finer splits reflecting more subtle variants of each class that reflect different developmental programs; for example, neocortical neurons are split into excitatory glutamatergic vs inhibitory GABAergic classes reflecting their different developmental origins in embryonic pallium vs subpallial proliferative regions, while the next splits in the GABAergic branch contain neurons generated by medial and caudal subdivisions of the ganglionic eminence and the preoptic area (Fig. 2a). These transcriptomic divisions are consistent with a long literature on cell fate specification of different GABAergic classes and the transcription factors involved in that process^62–64,67^ (Fig. 2b). Transcriptomics also allows quantitative analysis of developmental trajectories involved in this specification and maturation^62–64^ (Fig. 2c). Genes that differentiate neuronal classes are enriched for those involved in neuronal connectivity and synaptic communication, indicating they are predictive of selective cellular and circuit function^37^ (Fig. 2d). Finally, the same major transcriptomic classes of cortical GABAergic neurons are found in mammals and reptiles^68^ (Fig. 2e), suggesting deep conservation of cellular architecture and underlying mechanisms of molecular specification.

## Correspondence of cell-type classifications across modalities

Proposing a transcriptomic-based classification for a field traditionally centered on cellular anatomy, physiology and synaptic connectivity is challenging unless such a classification correlates strongly with those features. Recent work in the retina is promising in this regard, where a large body of work has established a highly diverse set of anatomically, physiologically and functionally discrete cell types^69^ and where transcriptomic clusters strongly correlate with this prior knowledge^35,69,70^. For example, for mouse bipolar cells, a class comprising 15 types of excitatory interneurons, there is essentially perfect correspondence between types defined by scRNAseq, high-throughput optical imaging of electrical activity, and serial section electron microscopy^35^. The spinal cord provides another good example of correspondence between scRNAseq and other cellular characteristics, including developmental origins and connectivity profiles^71,72^. Similarly, scRNAseq of mammalian hippocampus identifies neuronal cell types that were already described by anatomy and electrophysiology^73,74^.

**Fig. 3:**
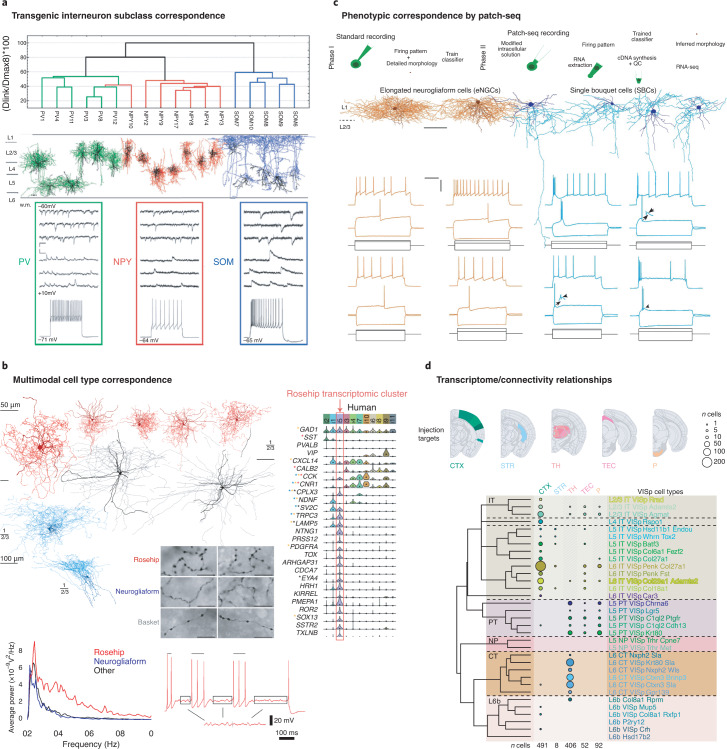
Correspondence across phenotypes of cortical neuron types. **a**, Quantitative morphological clustering and electrophysiological feature variation between major inhibitory neuron classes using transgenic mouse lines (modified from Figs. 1 and 2 from ref. ^31^). **b**, Convergent physiological, anatomical and transcriptomic evidence for a distinctive rosehip layer 1 inhibitory neuron type in human cortex that differs from neighboring neurogliaform cells. **c**, Morphological and physiological differences between layer 1 neurogliaform and single bouquet neurons shown by patch-seq analysis. Scale bars as in **b**. **d**, RNA-seq analysis of retrogradely labeled neurons in mouse primary visual cortex show distinctive projections of excitatory subclasses, but overlapping projections for finer transcriptomic cell types. Images in **a** adapted with permission from ref. ^31^, Oxford Univ. Press; in **b**–**d**, adapted with permission from refs. ^75,76^ and ^40^, respectively, Springer Nature.

Strong evidence for cross-modal correspondence in neocortical cell types is accumulating as well. An early application of cluster analysis of mouse layer 5 neurons showed correspondence between synaptic connectivity, morphology and even laminar position^13^. Almost perfect correlations were seen between major interneuron subclasses for molecular markers, axonal morphology and kinetics of synaptic inputs^31^ (Fig. 3a). Within somatostatin-positive interneurons, morphological and electrophysiological subgroups were correlated^22^. Other more specific neuron types show concordance between scRNAseq, physiology and morphology, such as the ‘rosehip’ cell, a layer 1 inhibitory neuron type in human cortex^75^ (Fig. 3b). Similarly, strong correspondence between scRNA-seq, electrophysiology and morphology was shown for mouse layer 1 neurogliaform and single bouquet neurons, using the patch–seq technique, which combines patch-clamp physiology and scRNA-seq^76^ (Fig. 3c). Finally, RNA-seq analysis of retrogradely labeled neurons in mouse primary visual cortex shows distinctive projections of transcriptionally defined excitatory subclasses^40^ (Fig. 3d). Experimental tools are increasingly available to aid in phenotypic characterization of transcriptionally defined cell types in model animals and even human, such as specific *Cre* lines and viruses, as well as novel spatial transcriptomics methods^54,77^. While major consortium efforts will generate the transcriptomic framework, linking different types of data to it will likely be most effective as a distributed community effort.

## Challenges for cortical cell type classification

Although strong cross-modal correspondence has been observed at the major subclass level, such correspondence at the more refined branches of the transcriptomic classification remains largely to be validated. One example is the already mentioned RNA-seq study of retrogradely labeled neurons in mouse primary visual cortex^40^. Despite distinct projection targets at the major branches of the transcriptomic taxonomy, there were overlapping projections for finer transcriptomic cell types (Fig. 3d). One possible explanation is that long-range connectivity patterns are set up early in development and may not be strongly reflected in adult gene expression. However, such mismatches do not negate the value of a core transcriptomic classification as described above. Rather, this information about developmental trajectories needs to be incorporated into the transcriptomic cell type classification^28^.

**Fig. 4:**
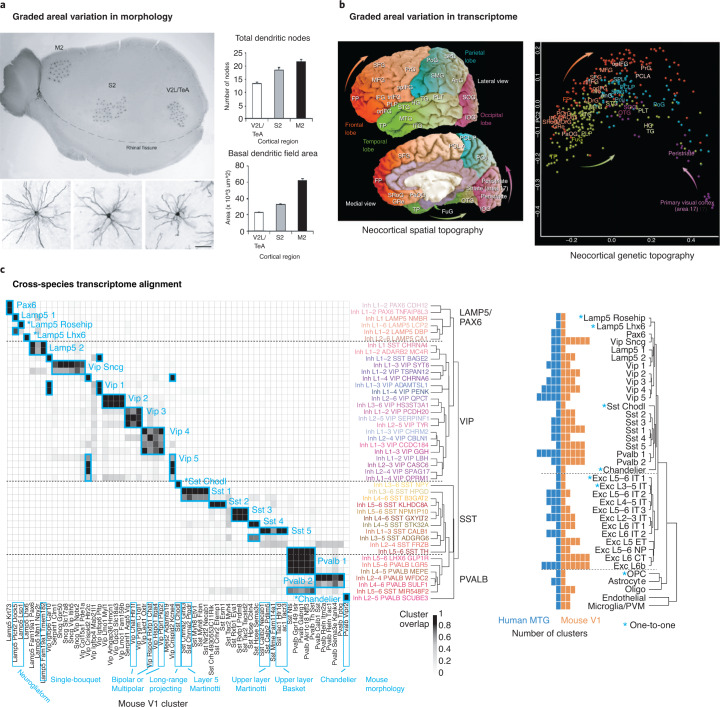
Challenges for transcriptomic classification. **a**, Gradients in morphological size and complexity across the rostrocaudal extent of the cortex. **b**, Graded transcriptomic variation across the human cortex encodes rostrocaudal position on the cortical sheet. **c**, Transcriptomic cell types can be aligned across species based on shared molecular specification, but often at a lower level of resolution than the finest types observed in a given species. Images in **a** adapted with permission from ref. ^82^, Oxford Univ. Press; in **b** and **c**, adapted with permission from refs. ^83^ and ^61^, respectively, Springer Nature.

Another challenge to transcriptomic classifications (and, in fact, to any classification of cell types) is the presence of phenotypic variation within a given cell type. One facet of this is the possibility of variation in gene expression due to cell state, differentiation and other dynamic processes within a single cell type. Some studies have suggested that cell types are possibly not defined, discrete entities and may be better described as components of a complex landscape of possible states^78–80^, and, indeed, some of that heterogeneity can be mapped with omics data^81^. Some continuous variation could be functionally relevant. For example, basal dendritic lengths and morphological complexity of layer 2/3 pyramidal cells appears to vary smoothly across a rostrocaudal axis in mouse cortex^82^ (Fig. 4a). Further evidence for spatial gradients can be found in the graded transcriptomic variation across the human cortex^83^, perhaps reflecting the expression of transcription factor gradients in the ventricular zone during development (Fig. 4b). These phenotypic or spatial gradients create challenges for thresholding in clustering, and they fuel debates between lumpers and splitters in determining the right level of granularity in defining cell types.

A particular advantage of a transcriptomic classification is that it provides a direct avenue for quantitative comparative analysis by aligning cell types across species based on shared gene covariation, enabling an ‘Ur-classification’ as a common denominator of basic cell types. For example, a recent study of human cortex^61^ demonstrated that the overall cellular organization of the human cortex is highly conserved with that of the mouse, allowing identification of homologous cell types (Fig. 4c). However, this study also revealed a challenge for the future, in that, in many cases, it was not possible to align cell types across species at the finest levels of granularity but rather at a higher level in the hierarchical taxonomy. Furthermore, many differences were seen in homologous types, including their proportions, laminar distributions, gene expression and morphology. Finally, prominent differences were found in non-neuronal cells as well, including astrocyte diversity and divergent molecular phenotypes between mouse and human that correlate with known morphological specializations in primate astrocytes^36,74,84^. Such similarities and differences between cell types across species, as well as challenges created by graded or developmental variations in features, could also be better captured by a probabilistically defined and hierarchically organized cell-type taxonomy.

## A probabilistic and hierarchical definition of cortical cell types

Examining the current transcriptomic evidence, in some cases we find highly distinct cell types based on robust similarities of the transcriptome and other measurable cell attributes, as exemplified by the phenotypic homogeneity of neocortical chandelier cells^40,85–87^ or the above-mentioned rosehip cells. On the other hand, the existence of cell states, spatial gradients of phenotypes and mixtures of differences and similarities in cross-species comparisons present challenges to a discrete and categorical perspective on defining cell types. Prematurely adopting an inflexible definition of types will obscure the significance of observed phenotypic variability and its biological interpretation. Rather, a plausible way forward is to employ a practical or operational quantitative definition of a cell type.

Cluster analysis has been used to classify cortical neurons according to their structural or physiological phenotypes or expression of molecular markers^13,14,22,31,82,87–90^ and, more recently, transcriptomics^36,40,91,92^. Many unsupervised and supervised methods can be used, including multilayer perceptrons^16^, logistic regression^16^, *k*-nearest neighbors^16^, affinity propagation^93^, Bayesian classifiers^34^, naïve Bayes^16^, topic modelling^94^, *t*-distributed stochastic neighbor embedding (t-SNE)^95,96^, graph theory^97^ and autoencoders^98^. These methods, building on the existence of statistically defined groups or clusters over a set of measurable attributes, naturally lead to an evidence-based probabilistic definition of cell types.

A probabilistic definition of cell types is particularly applicable to transcriptomics, where the dimension of the underlying space is large, the variance comparatively high and competing approaches give similar results. However, one requires community consensus on a rigorous statistical definition of transcriptomic types and the description of intra- and inter-type variability. Ideally, this quantitative definition of a cell type would be independent of the statistical method used (i.e., robust to different methods) and would include a description of quantitative metrics such as resolution, complexity, variability, uniqueness and association of variables with other attributes. There are two approaches to find and test cluster validity. One is ‘hard’ clustering, with clearly defined borders between clusters and with each cell strictly assigned to a particular type. Alternatively, in ‘soft’ (or ‘fuzzy’) clustering, any given cell has a particular probability of belonging to a particular cluster. Despite the probabilistic nature, inter- and intra-cluster distance may still be defined for outcome validation. Ultimately, the consensus description of cell types may form a continuum, beginning with hard and ending with soft distinctions among cell types, with an ambiguous transition between these extremes.

One natural approach to represent a transcriptomic taxonomy is to adopt a hierarchical framework. Cluster analysis is well suited to this, as its connectivity-based methods generate a tree-like representation of clusters^99^. This approach follows the historical tradition of using cladistics to classify organisms, assuming common ancestors in their evolution and synapomorphies (shared derived traits) among related clades. While statistical clusters do not presume any hierarchy in the structure of the data, biological systems have a temporal evolution as one of their essential features and makes temporally based hierarchies natural^100^. The evolutionary or developmental history of a neural circuit implies earlier stages, which are often less specialized and represent common ancestors of later states^101^. Indeed, a hierarchical organization of existing transcriptomic cell types data appears to mirror developmental principles and spatiotemporal organization in the neocortex (see above). Another advantage of casting the cell type classification as a cladistic one is that the lumping–splitting tension maps itself naturally as a distinction between different levels of the hierarchical tree, since one can split a group into subgroups at a lower level of the hierarchy to reflect data obtained in different physiological or developmental conditions. This provides an effective and objective framework to quantitatively evaluate lumper-vs-splitter discussions.

But hierarchical transcriptomic relationships may not be easily represented as a simple tree-like structure. Rather, they may have complex inclusion–exclusion and class relationships and may be more amenable to graph-based or other set-theoretic constructions. Indeed, the space of the transcriptomes for cortical cell types could be visualized as a complex, high-dimensional landscape with isolated peaks of expression for a given cell type but also valleys and gradients between more weakly defined classes, which could be described alternatively as types or states. Such complexity can be described using, for example, the concept of cell-type attractors^28^, or using the distinction between core and intermediate cells^40^ or the description of a cell type as a continuous trajectory in transcriptomic space^102^. A robust statistical framework that enables a quantitative definition of cell type (or tendency to be a type) is clearly needed.

A final, and key, question is how to ensure that any given classification or taxonomy is valid. The goal is not defining a classification system per se, but to create a comprehensive description of cellular diversity in the neocortex. One needs to ensure that the experimental method will indeed capture all of the cell types present, that the classification is complete and that the types are defined correctly. For any classification to be valid, it is critical to ensure accuracy and correctness. First, it is imperative to seek internal statistical robustness for identified clusters, using different statistical methods^22,103^. Second, external validation with orthogonal datasets is critical. Multimodal datasets are particularly important in this regard, as they enable cross-comparisons between classifications based on different types of data, for example, molecular, physiological or anatomical^22,31^, patch-seq^76^, or spatial transcriptomics methods^54^ (Fig. 3a–c) can enable this, defining functionally relevant levels of granularity. Finally, a probabilistic definition, particularly with a Bayesian framework, can be tested by generatively building computational models of each cell type and comparting them with the real data, thus providing some performance metrics on the algorithms. Using these criteria, robustness, reproducibility and predictive power can be measured and different approaches compared, as is normally done in machine learning^16^.

## A unified ontology and nomenclature of cortical cell types

To truly gain community adoption, the data-driven transcriptomic classification of cortical cell types requires a formal unified cell type classification, a taxonomy and a nomenclature system^17,20,90^ whose principles are generalizable to other systems. Names are important: as an old Basque proverb states, ‘*izena duen guzia omen da*’ or ‘that which has a name exists’, and a similar Chinese one says ‘the beginning of wisdom is to call things by their right names’. This classification should aim to be a consensus one that incorporates the richness of data accumulated by different groups and be presented in a curated output that is public, easily accessible and has revisions managed by a curation committee of experts. Creation of such an ontology is a serious project in data organization that can build on prior efforts in cell ontologies^104–106^, as well as best practices established by the ontology development community^107^ (see Open Biomedical Ontology Foundry, http://www.obofoundry.org).

A true, data-driven transcriptomic taxonomy poses a series of challenges that have not yet been taken on by the cell ontology community, but that are surmountable. One challenge is that transcriptomically similar cell types can exist in multiple anatomical locations. Thus, transcriptomic types need to be related to proper levels of the anatomical structure. Prominent gradients across cortical areas pose another challenge to define in a taxonomy. While any given cortical region contains some number of transcriptomic types, it seems likely that many of these types will vary in a somewhat continuous fashion across cortical areas and possibly also across species (Fig. 4a,b). Likewise, the classification system should also have a temporal component to capture the developmental trajectory from progenitor cell division to a terminally differentiated state. Cells can be quantitatively defined by their position on that developmental or spatial gradient. Finally, aligning across species is quantitatively possible now, but this alignment may only be possible at different levels of granularity with increasing evolutionary distance. The benefits of creating a unified reference ontology across these biological axes will be large, but it will be a serious community effort to design a system that can accommodate them.

Following the genetic classification paradigm proposed here, there are many lessons to learn from genomics. For example, the reference classification could be iteratively updated and refined with subsequent accumulation of data^108^ like genome builds, which changed in the early years but have become increasingly stable. As in current gene nomenclature, an official symbol with multiple aliases can link cell types to commonly used terminology relating to cellular anatomy or other phenotypes. This nomenclature should be portable across species, with orthologous cell types having common names, much as current gene symbols refer to orthologous genes. For the cell type classification to be useful like the genome has been, computational tools conceptually similar to BLAST alignment tools^109^ for mapping sequence data, need to be developed to allow researchers to quantitatively map their data to this reference classification. Finally, continuing the analogy with genomics, just as there are different versions of genome builds for different purposes (for example, with more or less manual curation), one could consider different versions of cortical cell type taxonomies, with varying levels of splitting or lumping; spatial, temporal or evolutionary criteria; or even some manually curated by experts, but under a unified framework of probabilistic definition of cell types.

Nomenclature also poses a challenge. Currently, the lack of standardized nomenclature makes it difficult to track and relate cell types across different studies. One natural idea with a genetically based paradigm is to name cell types on the basis of the best defining genes for each cell type, as is currently commonly done^36,61,110^. However, the most specific genes are not always detected in every cell of a cluster, and often the genes that best define a cell type in one species are not conserved in other species. The traditional way of naming cell types is by their anatomical features (such as chandelier, double-bouquet, basket, Martinotti, pyramidal cells), and it would be desirable to incorporate these short and widely-used names into a nomenclature when possible, to seek consistency with the vast literature on neocortical cell types. However, anatomical features, such as horsetail axons, may also vary across species^17^. Also, for newly identified cell types, anatomical information is often not available and naming them by marker genes will be more practical.

Adopting a more abstract nomenclature not based on anatomical features or individual marker genes could make it more flexible, more easily applicable across species and more compatible with other tissues outside the cortex or the brain. One idea for a cell-type nomenclature system is to build on gene nomenclature, treating transcriptomic cell clusters as sequence data (partially implemented for Allen Institute datasets; https://portal.brain-map.org/explore/classes/nomenclature). Every cell cluster from a dataset or analysis would get a unique accession ID. Robust and reproducible clusters would have official cell type names or symbols, as well as any number of aliases that could represent different existing nomenclatures or historical names. In addition to cell types, higher-order classes (for example, caudal ganglionic eminence (CGE)-derived GABAergic interneurons, GABAergic interneurons, neurons) could be named as well, and both types and classes would be matched across species at the level (type, class) at which they can be aligned.

## A cell-type knowledge graph for community data aggregation

Defining the cell types of the cortex (or other brain structures) serves as a foundation for aggregating information about their function. By analogy to the genome, the definition of genes has allowed a massive integration of information about their usage, function and disease relevance with a wide range of databases. On the other hand, probabilistically defined cell types are not the same as deterministically defined protein-coding regions of the genome, and we can expect that our understanding of cell types and their functional relevance will change as more information becomes available. A more flexible way to organize our knowledge and understanding of cell types would be as a living, updatable framework, one allowing reference, query and inference. An online-based data aggregation platform could also have a significant sociological impact in neuroscience by encouraging collaborative participation.

**Fig. 5:**
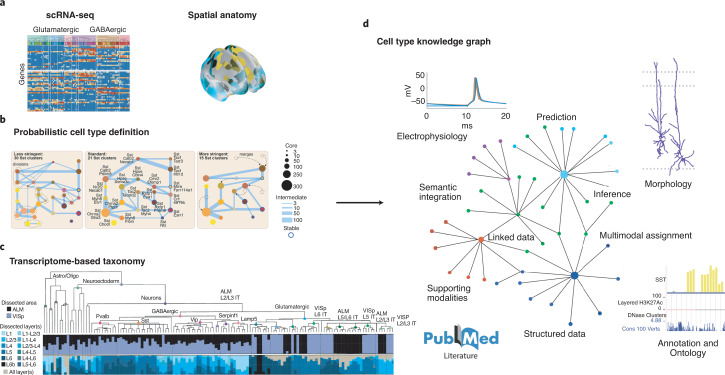
Transcriptome based taxonomy, probabilistic cell types, and cell-type knowledge graphs. **a**, A transcriptome-based cell-type taxonomy is constructed from scRNA-seq data, related epigenomic datasets and neuroanatomy, **b**, Cell types are initially defined based on transcriptomic signatures in a probabilistic manner with multiresolution clustering and statistical analysis to identify robustness and variability. **c**, Reproducible gene expression patterns identify hierarchies of putative cell types that are subject to further analyses and validation. **d**, Transcriptomic cell-type taxonomies form a basis for constructing cell-type knowledge graphs that summarize the present state of definable cell types. Multimodal assignment of data, such as morphology, electrophysiology and connectivity, is associated and reported with statistical variability over assigned types. A knowledge graph contains relevant and essential supporting information, such as supporting data for further analysis and mapping, descriptive annotation and ontology, and literature citations.

One example of an appropriate data structure for such a community platform is a ‘knowledge graph’, a widely used tool in the tech industry and computer science as a platform for data aggregation (Fig. 5). A knowledge graph is a relational data structure in which nodes represent entities (such as cell types and their attributes) and the links, or edges, between them represent their relational and statistical associations. There is a measurable graph-theoretic distance between nodes based on probable associations and known relationships. The cortical cell knowledge graph could be initialized with standardized transcriptomics data, after which other data modalities and related taxonomies could be readily mapped onto the graph to capture anatomical, electrophysiological, developmental and other cell properties. For example, important contributors to cell identity, determined by cellular interactions, splicing, local translation, protein phosphorylation, etc., may not be readily captured by scRNA-seq at present, but could be measured in future CAP datasets, which could then be added to the knowledge graph. In such a knowledge graph, there are two basic use-cases as new data becomes available. First, one can use it to identify known cell types and their properties in new datasets. With a probabilistic or Bayesian definition, each new cell will be assigned a probability of belonging to a particular type in the graph. Second, the graph can be manually or automatically updated, following conventional optimization algorithms, as new data can change node identities and distances with respect to one another.

The proposed cell-type knowledge framework would represent a living and updatable resource that maintains an actively derived and flexible ontology of cortical cell types, benefitting from present active ontology efforts. This standardized database could be powered by open-source algorithms and managed and curated by database administrators. It would be a dynamic database with query capability, but would only accept peer-reviewed published data in a standardized fashion and nomenclature, providing a common denominator for the research in the field, integrating quantitative and qualitative cell-type classification, and allowing for updates, subject to review and validation. Computational engines would allow new data to be compared and allow users to query the current state of cell-type understanding from the perspective of their new data, assigning the most likely type to multi- or unimodal datasets based on similarities to the current framework’s knowledge. In addition to supporting literature reference, the dynamic framework might include online forums for scientific discussion and education. Ultimately, a cell-type community knowledge framework would be a dynamic and living resource that researchers, clinicians and educators could refer to as the benchmark resource for cell types in the cortex, promoting collaborative participation in the field.

## Maintaining and updating the classification

The classification, nomenclature and associated knowledge graph could be managed by a committee of experts representing the breadth of approaches and disciplines in the field. Such a committee would be charged with designing the statistical classification model to sustain a basic taxonomy; the type of open platform to use for the knowledge graph; the rules by which this taxonomy can be updated and revised; the quality control or peer-reviewed criteria; and the metadata to be added. While the knowledge graph could continually update itself automatically, as new data is imported, different curated versions of the graph might be released in regular updates. This committee, arising from expert volunteers, could also help with vetting of a unified nomenclature of cortical cells that is succinct, useful and informative, as well as methods by which community input would be incorporated in a fair and efficient fashion.

Potentially, such a committee might be established and supported through existing organizations or consortia with interest in cell type classification, such as the NIH BRAIN Initiative Cell Census Network (BICCN; https://www.biccn.org), the NeuroLex–International Neuroinformatics Coordinating Facility (INCF; http://130.229.26.15/news/activities/our-programs/pons/neurolex-wiki.html), the Neuroscience Information Framework (NIF; https://neuinfo.org), the Human Brain Project (HBP; https://www.humanbrainproject.eu/), the Human BioMolecular Atlas Program (HuBMAP; https://commonfund.nih.gov/hubmap) or the Human Cell Atlas (HCA; https://www.humancellatlas.org/). Some of these groups are already chartered with mapping the cell types of the nervous system or other organs in the body and may have resources to build the backend technological infrastructure needed for the knowledge graph.

Regardless of who supports and maintains this key infrastructure, it is critical that the efforts be managed through open communication with the community. A public consortium will be a logical organizational structure for channeling diverse inputs and will also adequately represent the wider community, reflecting cultural, geographic, ethnic and gender diversity. Strong community engagement will ensure wide acceptance and ensure that these standards are adopted widely, within and outside of the neocortex specialist field.

## A community-based taxonomy and nomenclature of cortical cell types

To conclude, we think that the field of neocortical studies is ready for a synthetic, principled classification of cortical cell types, based on single-cell transcriptomic data and anchored on quantitative criteria that operationally define cell clusters based on their statistical and probabilistic grouping. Although molecularly driven initially, this taxonomy should be revised and modified as additional CAP datasets become available, becoming a true multimodal classification of cortical cell types. We view this core classification as potentially valid for all mammalian species and also as likely applicable to homologous structures in other vertebrates, as a broad framework to encapsulate evolutionary conservation with species specialization. Indeed, only with such a systematic approach to comparing cell types across species will it be possible to understand how cell type diversity evolved in the cerebral cortex.

This taxonomy will only be useful and successful if adopted by the community. So, in addition to the nomenclature, a series of research tools should be developed, ideally by a community consortium, to facilitate similar experimental access to these cell types by the broader range of investigators. We envision molecular and genetic tools, such as standard sets of antibodies and RNA probes to identify key molecular markers for each cell type, as well as cell or mouse lines that are used as resources for the entire community. Statistical tools to enable direct comparisons among datasets, and to enable mapping new datasets to reference datasets, are essential. An open informatics backbone needs to be developed as an essential part of the taxonomy, as well as visualization and analysis tools that take advantage of this taxonomy and allow scientists to explore the data, add to the knowledge base and achieve new knowledge.

In addition, we propose that the community input to support this taxonomy and enable its future revisions be channeled into an open platform, a knowledge graph, as is becoming increasingly common in community-led data science. Aggregation of knowledge through data graphs, now a common practice in the tech industry, will accelerate the dissemination of knowledge and could avoid the ‘publication graveyard’, where data are stored away in siloed journal articles disconnected from the rest of the field. Anchoring this taxonomy and knowledge graph, a unified new nomenclature of cortical cell types valid across species is needed to centralize efforts in the field, with a generalizable framework to integrate with other cell-type classifications. We view the establishment of a common nomenclature as an essential step to provide a standardized language that enables the meaningful aggregation and sharing of data.

If successful, this community-based classification effort, joined by a common nomenclature and nourished by the knowledge graph, could be extended and generalized to other parts of the brain or of the body. In this sense, the classification of neocortical cell types, a field with a long tradition and multidimensional approach to a central problem in neuroscience, could be an ideal test case to explore this novel organization of knowledge in neuroscience and, more generally, in biology.
