# Author Correction: A community-based transcriptomics classification and nomenclature of neocortical cell types

**DOI:** 10.1038/s41593-020-00779-0

**Published:** 2021-03-19

**Authors:** Rafael Yuste, Michael Hawrylycz, Nadia Aalling, Argel Aguilar-Valles, Detlev Arendt, Ruben Armañanzas, Giorgio A. Ascoli, Concha Bielza, Vahid Bokharaie, Tobias Borgtoft Bergmann, Irina Bystron, Marco Capogna, YoonJeung Chang, Ann Clemens, Christiaan P. J. de Kock, Javier DeFelipe, Sandra Esmeralda Dos Santos, Keagan Dunville, Dirk Feldmeyer, Richárd Fiáth, Gordon James Fishell, Angelica Foggetti, Xuefan Gao, Parviz Ghaderi, Natalia A. Goriounova, Onur Güntürkün, Kenta Hagihara, Vanessa Jane Hall, Moritz Helmstaedter, Suzana Herculano-Houzel, Markus M. Hilscher, Hajime Hirase, Jens Hjerling-Leffler, Rebecca Hodge, Josh Huang, Rafiq Huda, Konstantin Khodosevich, Ole Kiehn, Henner Koch, Eric S. Kuebler, Malte Kühnemund, Pedro Larrañaga, Boudewijn Lelieveldt, Emma Louise Louth, Jan H. Lui, Huibert D. Mansvelder, Oscar Marin, Julio Martinez-Trujillo, Homeira Moradi Chameh, Alok Nath Mohapatra, Hermany Munguba, Maiken Nedergaard, Pavel Němec, Netanel Ofer, Ulrich Gottfried Pfisterer, Samuel Pontes, William Redmond, Jean Rossier, Joshua R. Sanes, Richard H. Scheuermann, Esther Serrano-Saiz, Jochen F. Staiger, Peter Somogyi, Gábor Tamás, Andreas Savas Tolias, Maria Antonietta Tosches, Miguel Turrero García, Christian Wozny, Thomas V. Wuttke, Yong Liu, Juan Yuan, Hongkui Zeng, Ed Lein

**Affiliations:** 1 Columbia University, New York City, NY USA; 2 Allen Institute for Brain Science, Seattle, WA USA; 3 University of Copenhagen, Copenhagen, Denmark; 4 Department of Neuroscience, Carleton University, Ottawa, Ontario Canada; 5 European Molecular Biology Laboratory, Heidelberg, Germany; 6 George Mason University, Fairfax, VA USA; 7 BrainScope Company Inc., Bethesda, MD USA; 8 Universidad Politécnica de Madrid, Madrid, Spain; 9 Max Planck Institute for Biological Cybernetics, Tübingen, Germany; 10 University of Oxford, Oxford, UK; 11 Department of Biomedicine, Aarhus University, Aarhus, Denmark; 12 Department of Genetics, Harvard Medical School, Boston, MA USA; 13 The University of Edinburgh, Edinburgh, UK; 14 Vrije Universiteit Amsterdam, Amsterdam, Netherlands; 15 Cajal Institute, Madrid, Spain; 16 Vanderbilt University, Nashville, TN USA; 17 Scuola Normale Superior, Pisa, Italy; 18 Research Centre Jülich, Jülich, Germany; 19 Research Centre for Natural Sciences, Budapest, Hungary; 20 Harvard Medical School, Cambridge, MA USA; 21 Christian-Albrechts-University Kiel, Kiel, Germany; 22 European Molecular Biology Laboratory, Hamburg, Germany; 23 École Polytechnique Fédérale de Lausanne, Lausanne, Switzerland; 24 Ruhr University Bochum, Bochum, Germany; 25 Friedrich Miescher Institute for Biological Research, Basel, Switzerland; 26 Max Planck Institute for Brain Research, Frankfurt, Germany; 27 Karolinska Institutet, Stockholm, Sweden; 28 Science for Life Laboratory, Department of Biochemistry and Biophysics, Stockholm University, Solna, Sweden; 29 Cold Spring Harbor Laboratory, Laurel Hollow, NY USA; 30 WM Keck Center for Collaborative Neuroscience, Department of Cell Biology and Neuroscience, Rutgers University - New Brunswick, Piscataway, NJ USA; 31 Department of Neuroscience, University of Copenhagen, Copenhagen, Denmark; 32 RWTH Aachen University, Aachen, Germany; 33 Robarts Research Institute, Western University, London, Ontario Canada; 34 CARTANA, Stockholm, Sweden; 35 Leiden University Medical Center, Leiden, the Netherlands; 36 Stanford University, Stanford, CA USA; 37 King’s College London, London, UK; 38 Schulich School of Medicine and Dentistry, Departments of Physiology, Pharmacology and Psychiatry, University of Western Ontario, London, Ontario Canada; 39 Krembil Research Institute, Toronto, Ontario Canada; 40 University of Haifa, Haifa, Israel; 41 Univeristy of Rochester, Rochester, NY USA; 42 Charles University, Prague, Czech Republic; 43 Bar Ilan University, Ramat Gan, Israel; 44 Macquarie University, Sydney, New South Wales Australia; 45 Sorbonne University, Paris, France; 46 Harvard University, Cambrige, MA USA; 47 J. Craig Venter Institute, La Jolla, CA USA; 48 Department of Pathology, University of California, San Diego, CA USA; 49 Centro de Biologia Molecular Severo Ochoa (CSIC), Madrid, Spain; 50 Institute for Neuroanatomy, University of Göttingen, Göttingen, Germany; 51 University of Szeged, Szeged, Hungary; 52 Baylor College of Medicine, Houston, TX USA; 53 Department of Neurobiology, Harvard Medical School, Boston, MA USA; 54 University of Strathclyde, Glasgow, UK; 55 MSH Medical School, Hamburg, Germany; 56 Departments of Neurosurgery and of Neurology and Epileptology, Hertie-Institute for Clinical Brain Research, University of Tübingen, Tübingen, Germany

**Keywords:** Genetics of the nervous system, Neural circuits

Correction to: *Nature Neuroscience* 10.1038/s41593-020-0685-8, published online 24 August 2020.

In the version of this article initially published, multiple errors appeared in the author and affiliations lists. The errors have been corrected in the PDF and HTML versions of this article.

